# Urological Guidelines for Kidney Stones: Overview and Comprehensive Update

**DOI:** 10.3390/jcm13041114

**Published:** 2024-02-16

**Authors:** Mahir Akram, Victoria Jahrreiss, Andreas Skolarikos, Robert Geraghty, Lazaros Tzelves, Esteban Emilliani, Niall F. Davis, Bhaskar K. Somani

**Affiliations:** 1 Core Trainee in Urology, University Hospital Southampton NHS Foundation Trust, Southampton SO16 6YD, UK; mahirakram93@gmail.com (M.A.);; 2 Faculty of urology, University of Athens, 15772 Athens, Greecelazarostzelves@gmail.com (L.T.); 3 University Hospital Newcastle, Newcastle NE4 5NR, UK; 4 Fundacio Puigvert, 08025 Barcelona, Spain; 5 Tallaght Hospital, D24 NR0A Dublin, Ireland

**Keywords:** kidney calculi, ureteroscopy, PCNL, bladder calculi, laser

## Abstract

Background: Evidence-based guidelines are published by urological organisations for various conditions, including urolithiasis. In this paper, we provide guidance on the management of kidney stone disease (KSD) and compare the American Urological Association (AUA) and European Association of Urologists (EAU) guidelines. Methods: We evaluate and appraise the evidence and grade of recommendation provided by the AUA and EAU guidelines on urolithiasis (both surgical and medical management). Results: Both the AUA and EAU guidelines provide guidance on the type of imaging, treatment options, and medical therapies and advice on specific patient groups, such as in paediatrics and pregnancy. While the guidelines are generally aligned and based on evidence, some subtle differences exist in the recommendations, but both are generally unanimous for the majority of the principles of management. Conclusions: We recommend that the guidelines should undergo regular updates based on recently published material, and while these guidelines provide a framework, treatment plans should still be personalised, respecting patient preferences, surgical expertise, and various other individual factors, to offer the best outcome for kidney stone patients.

## 1. Introduction

Urolithiasis is a significantly common disease globally, despite the notable difference in the rate of occurrence based on various factors like gender, climate, diet, and other risk factors. There has been a yearly increase in the prevalence of stone occurrence in people above the age of 30 for all genders [1]. This rise in incidence, along with advances in modern technology, places a significant financial burden on healthcare facilities for the treatment of kidney stone disease (KSD), accounting for an estimated USD 5.3 billion spent on the disease globally in 2014, making it the second most costly urological ailment [2].

Extensive guidelines on urolithiasis have been published by various accomplished institutes throughout the world. Those developed by the American Urology Association (AUA) and the European Association of Urology (EAU) are widely accepted and used by clinicians worldwide to help them in the diagnosis, management, and follow-up of patients with kidney stone disease (KSD). Separate guidelines are published by the AUA for the medical and surgical management of KSD, which last saw an update in 2019 and 2016, respectively [3,4]. In comparison, the EAU provides a single document titled Urolithiasis for the management of urinary tract stones, with the latest update being in 2023 [5].

Separate methods are used by both guidelines to assess the strength of evidence used in the guidelines. The AUA guidelines use letters for grading, namely A, B, and C, depending on the quality and certainty of the evidence [6]; this is followed by certain nomenclature that differs for medical and surgical guidelines. The surgical guidelines use statements of strong, moderate, or conditional recommendations, whereas the medical guidelines use nomenclature reflecting the options, recommendations, and standards, all of which are based on the risk–benefit ratio for patients. Cases where evidence is lacking, clinical principles, and expert opinions are used to provide additional information.

In contrast, the EAU classifies its recommendations as “strong” or “weak”, using the guiding concept of the GRADE methodology [7], based on various factors, such as the quality and extent of the effect, certainty, balanced outcomes, and patient values and preferences [8]. The EAU guidelines also outline goals that the panel aims to achieve for the 2024 update, which are aimed at further evaluating evidence for efficient practice in endourology and questioning the accuracy of the stone size in selecting treatment options.

While guidelines provide valuable information and clinical frameworks by consolidating the best available evidence, they can never guarantee the best results for patients, especially due to limitations in their upgradation [8]. Hence, when formulating a treatment plan, clinical expertise and individual patient circumstances should always be the foremost factors under consideration, and guidelines should never override these.

## 2. Initial Assessment

### 2.1. Presentation and Evaluation

Urolithiasis can present a variety of symptoms, ranging from fever, vomiting, and loin pain to being completely asymptomatic. Bladder stones can present as recurrent urinary tract infections (UTIs), urinary frequency, terminal haematuria, or suprapubic pain. A detailed medical history review and a physical examination should be carried out during presentation. The EAU recommends expediting the investigation if there is a lack of understanding of the diagnosis, pyrexia, or a solitary kidney (strong recommendation). However, the imaging modality should not delay effective analgesia and resuscitation. 

### 2.2. Investigations

#### 2.2.1. Renal and Ureteric Stones

The EAU recommends ultrasound (US) as the initial investigation in asymptomatic patients as it is safe, less costly, and can identify hydronephrosis and calculi in the renal calyces, pelvis, and pelvic-ureteric and vesico-ureteric junctions. Non-contrast-enhanced computed tomography (NCCT) is the investigation of choice in symptomatic patients as it can classify the stone density, diameter, volume, distance from the skin, and the adjacent anatomy, all of which can help in selecting from the treatment options available (EAU: strong recommendation). It is appreciably more precise than US or an intravenous urogram (IVU) [9]. If the assessment of the anatomy of the collecting system is required before stone removal, contrast-enhanced imaging should be performed (EAU: strong recommendation). The EAU also suggests that although kidney ureter bladder (KUB) X-ray can differentiate the radiopacity of stones, they need not be performed if NCCT is being considered.

The AUA also recommends NCCT as the imaging method of choice for the assessment of patients with urolithiasis prior to percutaneous nephrolithotomy (PCNL) (AUA: strong recommendation) and also for treatment selection between shock wave lithotripsy (SWL) and ureteroscopy (URS) (AUA: conditional recommendation), and it discourages the use of US solely for this purpose. If significant kidney injury is suspected, functional imaging modalities such as a diethylene-triamine-penta-acetate (DTPA) or mercaptoacetyltriglycine (MAG-3) renogram can be used to help guide treatment (AUA: conditional recommendation).

Besides imaging, various haematology, serum biochemistry, and coagulation tests should also be performed. The EAU recommends serum creatinine, uric acid, ionised calcium, sodium, potassium, blood cell counts, C-reactive protein, and coagulation tests if an intervention is planned (EAU: strong recommendation). Both the EAU and AUA strongly recommend a urine culture and microscopy before any intervention.

#### 2.2.2. Bladder Stones

Symptomatic patients presenting with suspected bladder stones should undergo US as the first imaging modality; however, if the patient remains symptomatic, with US being inconclusive, NCCT or cystoscopy should be considered, given the higher sensitivity of both for diagnosis when compared to US [10] (EAU: strong recommendation). X-ray KUB, despite providing useful information on radiopacity, has low accuracy for stone detection [11]; hence, there is a weak recommendation to use it in treatment planning and the follow-up of patients (EAU: weak recommendation). Given the sparseness of evidence, there are no specific guidelines on imaging modalities for children with suspected bladder stones. The AUA does not provide any guidelines on the diagnosis of bladder stones.

## 3. Available Treatment Options and Their Specific Considerations

### 3.1. Medical Treatment

Several drugs are available to be used as medical expulsive therapy (MET) for urolithiasis [3,4,5]. These include α-blockers, calcium channel inhibitors, and phosphodiesterase type 5 (PDE5) inhibitors, and, although an off-label indication, a class effect of α-blockers has been demonstrated as MET through various meta-analyses [5]. α-blockers should be considered for distal ureteral stones >5 mm (EAU: strong recommendation), and distal ureteral stones ≤10 mm should be offered α-blockers (AUA: strong recommendation).

### 3.2. Oral Chemolysis

Oral chemolitholysis, based on the alkalinisation of the urine by alkaline citrate or sodium bicarbonate, can be used to dissolve uric acid stones. Although this treatment has been used for a long time, no randomised controlled trials (RCTs) are available [5]. Potassium citrate should be offered for the alkalinisation of urine to patients with uric acid and cystine stones. However, favourable outcomes in the form of stone dissolution are inconsistent (AUA: expert opinion). Patients undergoing this treatment should be monitored during and after chemolysis, be informed on how to check the urinary pH, and monitored to alter the drug dose accordingly (EAU: strong recommendation).

### 3.3. Extracorporeal Shock Wave Lithotripsy (ESWL)

SWL, although having a lower stone-free rate (SFR), is associated with fewer overall complications than other endourology procedures, like ureteroscopy (URS) and PCNL [12,13]. Factors determining the efficacy of SWL include the patient’s habitus; the size, location, and composition of the stone; and the performance of SWL [5]. An appropriate coupling agent, such as an ultrasound gel, should be used to prevent the deflection of shock waves, along with meticulous radiological monitoring with either fluoroscopy or US during the procedure (EAU: strong recommendation). Adequate analgesia should be prescribed as it improves the outcome by limiting pain-induced movements (EAU: strong recommendation). Antibiotics should be prescribed in the case of infected stones or laboratory evidence of infection before any intervention (AUA/EAU: strong recommendation). The AUA strongly recommends not routinely placing stents in patients undergoing SWL; the EAU also advises no improvement in SFR with routine stenting, although it might reduce the formation of steinstrasse. α-blockers can be prescribed following SWL to ease the passage of stones, after informing the patient of it being an off-label indication (AUA: moderate recommendation), while the EAU also recommends the same when using MET after SWL. The EAU lists certain contraindications to SWL, which include uncontrolled UTIs, severe skeletal malformations and obesity, pregnancy, bleeding disorders, anatomical obstructions distal to the stone, and an arterial aneurysm close to the stone.

### 3.4. Ureteroscopy (URS)

URS is associated with a higher SFR and a better clinical outcome when compared to SWL [14]. The morbidity and complications of the procedure have also been appreciably better in the current endourological times [15]. Pre-procedural stent placement is not recommended by either the AUA (AUA: strong recommendation) or the EAU [4,5]. The AUA suggests that pre-stenting may be associated with a higher SFR and shorter operative time. However, in the absence of high-level evidence, the panel advocates against this practice. Following the intervention, if the patient is not at an increased risk of complications post-procedure, stenting should be avoided due to its association with higher morbidity and not being cost-effective (EAU/AUA: strong recommendation). If stented, both guidelines agree on the administration of α-blockers to reduce stent discomfort (EAU: strong recommendation, AUA: moderate recommendation). Stone removal should always be done under direct visualisation of the stone (EAU: strong recommendation), and a safety guide wire used where possible (AUA: expert opinion). The EAU advises on the use of a ureteral access sheath (UAS) if expecting a long operating time or when encountering large and multiple renal stones [16]. Being effective in all stone types, the EAU recommends using the holmium:yttrium–aluminium–garnet (Ho:YAG) laser for URS (EAU: strong recommendation); however, while the results of the thulium fibre laser (TFL) seem to be equivalent to those of Ho:YAG, more comparative clinical studies are currently needed between these modalities [5]. Both guidelines agree on the use of peri-operative prophylactic antibiotics before any endoscopic procedure. The EAU also advocates for the use of percutaneous antegrade URS if SWL has failed and retrograde URS is not an option for ureteral stones, and the use of flexible URS if PCNL and SWL are not an option, even for stones > 2 cm (EAU: strong recommendation). URS is also the treatment of choice in cases where stone removal is required without discontinuation of antithrombotic therapy (EAU/AUA: strong recommendation). Apart from general complications associated with anaesthesia and untreated UTIs, the EAU documents URS to be safe for the majority of patients without any particular contraindications [5].

### 3.5. Percutaneous Nephrolithotomy (PCNL)

PCNL is the first-line procedure for most large renal calculi, as it offers the advantage of a higher SFR since the effectiveness of this method is not limited by the stone burden or composition [17,18]. Before the procedure, essential imaging should be available to delineate the anatomy of the collecting system and surrounding structures, to ensure a safe percutaneous path to the renal stone. This can either be a US or a CT scan (EAU: strong recommendation), and the AUA also strongly recommends obtaining an NCCT before a PCNL. The EAU advises patient positioning to depend on the surgical competence and equipment available, with both prone and supine positions being equally safe, and the use of small instruments with mini-PNL (mPNL 12-22Fr) is associated with a shorter hospital stay, less blood loss, and an SFR similar to that of a standard PCNL (>22Fr) [5]. To allow the removal of fragments from areas not accessible by a rigid nephroscope, flexible nephroscopy should be a standard part of PCNL. To prevent electrolyte derangements, normal saline should always be used as an irrigation solution for PCNL and URS (AUA: strong recommendation). If the procedure has been uncomplicated, a tubeless PCNL (i.e., without nephrostomy) or totally tubeless (i.e., without nephrostomy and ureteral stent) should be considered, as it is linked with a shorter hospital inpatient stay and better pain control postoperatively (EAU: strong recommendation, AUA: conditional recommendation). A significant change highlighted by the EAU guidelines compared to the last version includes collecting a urine or stone culture directly from the renal pelvis during PCNL (EAU: strong recommendation). This is based on the fact that sepsis can occur during or after the procedure, even with a sterile pre-op urinary culture. Hence, cultures taken directly from the pelvis can more accurately anticipate this episode [19,20] and guide antibiotic therapy by recognising the causative organisms. The EAU mentions contraindications of PCNL as follows: tumour in the access tract area, malignant renal tumour, pregnancy, untreated UTIs, and anti-coagulant therapy, which must be carefully monitored and discontinued pre-operatively [5].

### 3.6. Open Surgery and Laparoscopy

Developments in endourology have led to open and laparoscopic approaches for the treatment of urolithiasis being rarely used. Both guidelines agree strongly on offering open or laparoscopic approaches for stone removal only when SWL, URS, and PCNL are unlikely to provide a decent opportunity for stone removal and more likely to fail (EAU/AUA: strong recommendation). The AUA mentions patients with stones and anatomical defects requiring reconstruction as one of the cases where an open or laparoscopic approach may prove to be more beneficial than endourology [4]. 

## 4. Management

### 4.1. Renal Colic

Ureteral stones causing acute renal colic usually present as an emergency that requires immediate and adequate analgesia [21]. Paracetamol and non-steroidal anti-inflammatory drugs (NSAIDs) can prove to be very effective in this instance and provide better pain relief than opioids; hence, they should be used as a first-line drug after ruling out contraindications. They also reduce inflammation and prevent pain relapse in patients being managed conservatively (EAU: strong recommendation). Pain persisting despite analgesia warrants renal decompression and endoscopic stone removal (EAU: strong recommendation).

### 4.2. Obstruction, Sepsis, Anuria

Sepsis secondary to an infected obstructed system accounts for major mortality and morbidity related to urolithiasis and its treatment [22,23]. Both the EAU and AUA strongly recommend urgent decompression with either percutaneous nephrostomy (PCN) or ureteric stenting, with none proving to be superior to the other, delaying any definitive treatment until the resolution of sepsis. Antibiotic treatment should not be delayed; it can be modified later once sensitivities have returned. Intensive care input should be sought if required, and a urine sample collected at the time of decompression (EAU: strong recommendation).

### 4.3. Ureteric Stones

#### 4.3.1. Conservative Management/Medical Expulsive Therapy (MET)

A policy of wait and watch/observation with regular evaluation can be applied to patients without complications due to stone disease [24]. The EAU does not specify any stone size and refers to the stone size as small, but it highlights that stone expulsion spontaneously is inversely proportional to the stone size and is different for every patient [25] (EAU: strong recommendation). In contrast, the AUA specifies a stone size of ≤10 mm for this policy (AUA: strong recommendation).

Both the AUA and EAU recommend the use of an α-blocker as MET for distal ureteric stones; the AUA suggests this for stones of ≤10 mm, whereas the EAU implies this for stones of <5 mm in size (AUA/EAU: strong recommendation). The AUA suggests that this treatment should be carried out for 4–6 weeks, and, following this, if not successful, a definitive treatment plan should be formulated (AUA: moderate recommendation). The EAU does not recommend any specific interval between MET and definitive treatment.

#### 4.3.2. Active Treatment

Active stone removal is warranted by either shock wave lithotripsy (SWL) or ureteroscopy (URS) for the following patients [5]:

- Stones unlikely to pass spontaneously;
- Urolithiasis causing obstruction;
- Pain despite adequate analgesia;
- Decreased renal function either due to renal failure, solitary kidney, or bilateral obstruction.

Reimaging in the form of X-ray KUB, US, or CT should be considered before any intervention if the initial presentation has changed, as this might indicate a change in the position and hence modified management is necessary (AUA: clinical principle). Patients undergoing intervention should be informed by the clinician about URS being associated with a higher stone-free rate (SFR) and lesser need to reperform the procedure when compared to SWL [26]; however, SWL is safer, with fewer complications and lower morbidity, in comparison with URS (AUA/EAU: strong recommendation).

The EAU recommends URS as the first-line treatment for stones either in the proximal or distal ureter measuring >10 mm; for <10 mm, either URS or SWL can be selected for first-line management (Table 1). Severe obesity also warrants URS as a first-line treatment option (EAU: strong recommendation). The AUA suggests offering URS to all patients requiring intervention with stones in the mid- and distal ureter due to a higher SFR, particularly with a stone size <10 mm, and SWL should be offered if the patient declines URS (AUA: strong recommendation). The AUA recommends that this guideline does not apply to stones in the proximal ureter as the SFR with URS for proximal ureteric calculi > 10 mm was not found to be superior to that of SWL based on the panel’s analysis.

### 4.4. Renal Stones

#### 4.4.1. Conservative Management

Due to advances in and the increased use of imaging technology, it is becoming easier to detect asymptomatic renal stones. Patients presenting with non-problematic stones in the calyces, which are not causing obstruction, can be offered observational treatment (AUA: conditional recommendation). This depends on the natural history of non-obstructing small renal calculi and the risk of progression, which are both unclear and not well defined [5]. Hence, this treatment option remains debatable due to the lack of high-quality evidence according to both the AUA and EAU. Both guidelines suggest active surveillance if opting for conservative treatment. While the AUA does not provide any specific timeframe, the EAU recommends 6 months for the first follow-up and yearly thereafter to assess for symptoms and stone growth with US or X-ray. If considering intervention, then NCCT should be used as the imaging modality (EAU: strong recommendation). 

#### 4.4.2. Active Treatment

Depending on the patient’s history and any previously available stone analysis, or the Hounsfield Units (HU) of the current stone on a CT scan, the stone’s composition should be assessed before offering any active treatment, as SWL is unlikely to be effective for stones with a density of >1000 HU (EAU: strong recommendation) 

#### 4.4.3. Renal Pelvis or Upper/Middle Calyces Calculi

Stones measuring >20 mm should be treated with PCNL as the first-line treatment (AUA/EAU: strong recommendation) (Table 2). This is due to the higher SFR with PCNL and lesser need for secondary procedures. Moreover, the effectiveness of PCNL when compared to SWL and URS is less affected by the stone’s location, density, and composition [4]. If PCNL is not available as an option, flexible URS or SWL should be considered (EAU: strong recommendation). Owing to the acceptable SFR with URS and SWL for <20 mm stones, they should be offered as first-line treatments for <20 mm calculi in this anatomical location (AUA: strong recommendation). The EAU suggests either SWL, URS, or PCNL for 10–20 mm stones; for <10 mm, it recommends SWL or URS as the first line, followed by PCNL.

#### 4.4.4. Lower Pole Calculi

The guidelines of the AUA and EAU for >20 mm lower pole calculi remain the same as those for upper and middle pole calculi. For ≤10 mm stones, they consider SWL or URS as the first line (AUA: strong recommendation). The EAU also suggests SWL or URS initially and PCNL as a second line. The guidelines slightly differ for calculi between 10 and 20 mm. Owing to the considerable benefit of the endoscopic approach over SWL, the AUA advocates strongly against offering SWL as a first line for 10–20 mm stones and to inform patients of the higher SFR with PCNL but also the associated increased morbidity (AUA: strong recommendation). The EAU describes favourable and unfavourable factors for SWL consideration; if adverse factors are present, it recommends PCNL (EAU: strong recommendation). However, in the presence of favourable factors, SWL can be considered alongside PCNL and URS as a first-line procedure. The EAU lists a narrow infundibulum, a long skin to stone distance, a long calyx, stones that are shock-wave-resistant (calcium oxalate monohydrate, brushite, cystine), and a steep infundibular-pelvic angle as factors that are unfavourable for SWL [5].

#### 4.4.5. Bladder Stones

Migratory bladder stones (those that have passed from the upper urinary tract) can be treated conservatively, especially if asymptomatic. However, primary (i.e., forming in absence of any other urinary tract abnormality) and secondary (i.e., occurring in the presence of other urinary tract pathologies) ones are less likely to pass spontaneously and require active management [5]. Several treatment options for bladder calculi are available. Of these, transurethral, endoscopic, and percutaneous treatments have similar SFRs but offer the advantage of a shorter duration of surgery, hospital stay, and catheterisation when compared to open cystolithotomy [27]. Transurethral cystolithotripsy should therefore be offered to adults as a first line and, where not possible, a percutaneous cystolithotripsy should be considered (EAU: strong recommendation). Open cystolithotomy can be used as a first line for very large bladder stones (EAU: weak recommendation). When endoscopic techniques are not preferable, laparoscopic, SWL, or open cystolithotomy approaches can be used as alternative options (EAU: weak recommendation), although the guidelines highlight the fact that SWL is associated with a lower SFR for bladder calculi compared to open or endoscopic procedures.

## 5. Specific Patient Groups

### 5.1. Paediatric Population

Urolithiasis in children can present with ill-defined symptoms, which can vary with age. Older children can present clinically with haematuria, flank pain, and recurrent UTIs [28], whereas, in infants, the symptoms can be vomiting, irritability, and crying [29]. 

Keeping in mind the effects of radiation and observing the principle of ALARA (As Low As Reasonably Achievable), US should be performed as primary imaging in the paediatric population with suspected stones; however, if it fails to provide adequate information, X-ray KUB or NCCT should be considered (EAU: strong recommendation). The AUA suggests NCCT before PCNL in children (AUA: strong recommendation).

The EAU documents a paucity of evidence for the observational management of stones in children; however, it can be the first approach for asymptomatic lower pole, <7 mm stones not composed of struvite or cystine and with no anatomical abnormalities [5]. It also suggests that although MET can increase the expulsion rate and control pain, it is associated with more side effects in children. Conversely, the AUA recommends conservative management with or without the use of MET as a first line for ≤10 mm ureteral stones [3], and, if MET is being used, parents or patients should be informed about the off-label indication (AUA: moderate recommendation). Stenting should not be routinely carried out prior to URS in children (AUA: expert opinion). Indications for SWL, URS, and PCNL are similar to those in adults, and SWL should be offered as a first line for single ureteral stones <10 mm, followed by URS as a second line if SWL fails or is not feasible (EAU: strong recommendation). Ureteroscopy and lasertripsy is a safe option with good outcomes provided that it is carried out by surgeons experienced in managing the complications associated with the procedure in the paediatric population [30]. The AUA advises either SWL or URS depending on the anatomy and body habitus with a failed observational strategy or a trial of MET; however, it suggests that the use of SWL may be restricted in this instance given the inability of ultrasound-based lithotriptors to accurately visualise the ureters, especially the mid-ureter, but it can prove more effective in specific groups of patients, such as small children, where their anatomy makes URS access more difficult (AUA: strong recommendation). Cases with renal calculi <20 mm should be offered SWL as a first line (EAU: strong recommendation). Flexible ureteroscopy (FURS) can be utilised if SWL fails, especially for lower calyceal stones [31]. The AUA agrees with using either SWL or URS for <20 mm renal stones (AUA: moderate recommendation). For renal stones >20 mm, PCNL should be offered (EAU: strong recommendation). PCNL, while offering a higher SFR, is associated with greater side effects in the paediatric population; however, the use of mini and ultra-mini PCNL techniques has led to a fall in complications, particularly those associated with the size of the tract, such as renal extravasation and haematuria [32]. However, the AUA differs slightly by advocating for both SWL (with stent or nephrostomy placement) and PCNL, being acceptable options for >20 mm renal calculi (AUA: moderate recommendation). The AUA also suggests the conservative treatment of non-obstructing, asymptomatic renal stones with regular routine US surveillance (AUA: expert opinion). Stone material should be analysed in all children, followed by a metabolic assessment depending on the results (EAU: strong recommendation).

### 5.2. Pregnancy

Urolithiasis in pregnancy can present a challenging clinical scenario and should be managed with a multidisciplinary approach involving radiologists, urologists, obstetricians, and the patient [33,34]. 

For pregnant patients, although NCCT has a higher positive predictive value than magnetic resonance imaging (MRI) and US, it should still be used as the last line, with US being the initial investigation, followed by MRI (EAU: strong recommendation). The AUA recommends a multidisciplinary approach before considering any diagnostic imaging in pregnant patients (AUA: clinical principle).

Where possible, uncomplicated and asymptomatic nephrolithiasis in pregnancy should be treated with an observational approach (AUA/EAU: strong recommendation). If conservative treatment fails, URS should be considered, or temporary decompression with ureteral stent or nephrostomy; however, they do require frequent exchanges due to rapid encrustation (AUA: strong recommendation). The EAU documents that URS (when compared to a temporary stenting) is associated with better patient satisfaction, lesser irritation of the urinary tract, and a reduced requirement for ureteric stent exchange [5].

### 5.3. Renal Transplant Patients

Although uncommon, stones in transplanted kidneys can present a difficult and clinically challenging scenario. They can arise as de novo allograft urolithasis or can be present in the donor kidney before transplant and can be detected by US or NCCT if US is inconclusive [5]. Obstruction secondary to stones in transplant patients requires immediate and effective treatment as they are dependent on the solitary kidney for the maintenance of renal function. The EAU suggests offering patients any of the available treatment options, SWL, URS, or PCNL, depending on various factors including, but not limited to, anatomical variations in the transplanted kidney, the transplant function, and the clotting status of the patient (EAU: weak recommendation). The treatment of donor stones prior to transplant may be required as it increases the donor pool and also because calculi in a donor kidney are considered a relative contraindication to donation. Ex vivo stone surgery, also known as ex vivo bench surgery, including ureteroscopy or pyelolithomy, can be carried out immediately after donor nephrectomy and has been proven to be safe, without any effect on the allograft’s function [35]. In addition, URS performed at experienced centres has also been demonstrated to be a safe technique, with good outcomes for both donor and post-transplanted kidneys [36]. Finally, SWL, although infrequently utilised for de novo lithiasis in transplant patients, has also been proven to be potent, with a low risk of major complications [37].

### 5.4. Post-Procedural Imaging

Following treatment with endourology or SWL, there may be residual fragments, which may require additional treatment [4]. The EAU recommends performing imaging following these interventions (EAU: strong recommendation). Due to the sparsity of high-quality evidence, the EAU suggests that the timing for imaging and the decision for the treatment of stone fragments should be based on the judgment of the treating clinician in conjunction with patient preferences. The EAU also advises that imaging within 4 weeks may lead to false positive results due to dust particles and consequently lead to overtreatment. NCCT has higher sensitivity to detect these fragments when compared with X-ray, US, and IVU; however, this decision must be based on the risks and benefits considering that NCCT is associated with increased radiation exposure and the detection of clinically insignificant particles [5]. The AUA advocates for considering endourological procedures if residual fragments are detected, and especially in the presence of infected stones (AUA: moderate recommendation).

### 5.5. Recurrence Prevention and Metabolic Evaluation

Following a new diagnosis of urolithiasis, all patients will need further metabolic work-up. Hence, an initial screening evaluation should be performed, which should include a detailed medical and nutritional history, drug history, urinalysis including dipstick and microscopy, and a serum blood sample including parathyroid hormone levels (PTH) if suspecting hyperparathyroidism (AUA: clinical principle). Once the stone has passed, patients can be classified as being of high or low risk for stone formation depending on the basic screening evaluation and stone analysis [5]. Therefore, when possible, all first-time stones should be sent for stone analysis with X-ray diffraction or infrared spectroscopy (EAU: strong recommendation, AUA: clinical principle). A repeat analysis is warranted for the early or late recurrence of stones or recurrence despite being on medical therapy (EAU: strong recommendation). 

Patients falling into the high-risk group require metabolic assessment as they are likely to benefit from medical therapy. This can be extended to first-time stone formers as well for nutritional guidance or even for drug therapy if applicable (AUA: standard). Specific dietary therapy based on the metabolic evaluation results is more effective than general dietary guidance to prevent recurrence [38].

One or two 24-h urinary samples should be collected for metabolic assessment, with the AUA panel preferring two samples. When the patient is on a random diet, this should be assessed at least for pH, volume, calcium, uric acid, sodium, citrate, oxalate, potassium, and creatinine (AUA: expert opinion). The EAU also favours the collection of two consecutive 24-h urinary samples. It also mentions spot urine samples, although of limited use, but they can be an alternative when 24-h collection is not possible (for example, in children) [5]. Ideally, the patient should be stone-free for at least 20 days and on their normal regular diet before the initial metabolic assessment [39].

Both guidelines provide dietary and medical management advice depending on the metabolic assessment and stone analysis results. The EAU also provides general preventive measures, including lifestyle, fluid, and general nutritional advice for a balanced diet. The EAU and AUA both stress the importance of fluid intake, highlighting the fact that the risk of stone formation is inversely related to high fluid intake, and recommend generous fluid intake to allow the 24-h urinary volume to be at least >2.5 L (EAU: strong recommendation, AUA: standard).

### 5.6. Calcium Oxalate Stones

The most common metabolic abnormalities associated with calcium stone formation include hyperoxaluria, hyperuricosuria, hypocitraturia, hypomagnesuria, and hypocalciuria [5] (Table 3). Recurrent calcium stones in the absence of abnormalities in a 24-h urinary sample should be treated with potassium citrate and/or thiazide diuretics (AUA: standard).

### 5.7. Uric Acid and Urate-Containing Stones

The recurrence rate in uric acid and ammonium urate stone formers is considered to be high [5] (Table 4). Uric acid stones are commonly associated with a low urinary pH or hyperuricosuria. Ammonium urate stones are rare and are associated with malnutrition, UTIs, hypokalaemia, hypophosphatemia, and malabsorption [5]. The urinary pH should be raised by administering potassium citrate, and persistent alkalinisation can also help to dissolve existing stones [40,41]. Allopurinol should not be offered as a first line to uric acid stone formers (AUA: expert opinion). The EAU strongly recommends using allopurinol in the case of high urinary uric acid levels (EAU: strong recommendation).

### 5.8. Cystine Stones

The main management strategy for cystine stones includes maintaining the pH of urine > 7.5 along with dietary therapy [42,43] (Table 5). Since the formation of cystine stones is based on the concentration of cystine, high fluid intake is essential [3], and the AUA recommends at least 4 L/day and the EAU suggests 3.5 L/day. Sodium and protein intake should be limited, and potassium citrate is used as a drug therapy for alkalinisation (AUA: expert opinion). The EAU agrees on the use of potassium citrate and also recommends tiopronin in cases where alkalinisation and dietary therapy are not effective (EAU: strong recommendation).

### 5.9. Calcium Phosphate Stones

Brushite and carbonate apatite are two different mineral forms in which calcium phosphate stones can appear; while brushite is associated with hypercalciuria and hyperphosphatemia, carbonate apatite can occur due to infections [5] (Table 6). Therefore, calcium phosphate stones can be seen in UTIs, hyperparathyroidism, and renal tubular acidosis. The EAU strongly recommends prescribing thiazides in the case of hypercalciuria (EAU: strong recommendation)

### 5.10. Struvite Stones

Stones containing struvite can either form from scratch or from the infection of pre-existing stones by urease-producing bacteria [44,45] (Table 7). In either case, the first-line treatment is complete surgical removal and antibiotic prescription in the case of persistent bacteriuria (EAU: strong recommendation). The AUA recommends offering acetohydroxamic acid (AHA), which acts as a urease inhibitor, when the surgical options are exhausted (AUA: expert opinion).

## 6. Areas of Future Research

Future studies should include costs and patient-reported outcomes in their reports. Similarly, newer technological innovations should be included with regular updates, with new lasers, day-case surgery, smaller scopes, artificial intelligence, and suction devices. Standardised definitions and reporting should be encouraged to help to compare outcomes, improve patient counselling, and inform decision making.

## 7. Conclusions

Kidney stone disease is a worldwide prevalent disease, and, due to various factors, especially diet- and climate-related, the prevalence across all ages, races, and sexes is showing an upward trend. At the same time, endourology is undergoing constant evolution, which in turn will alter the future management of urolithiasis. Guidelines like those published by the AUA and EAU cover an extensive area and use a high-quality, evidence-based approach towards helping clinicians to tailor specific plans for the management of stone disease. Despite the minor differences between the AUA and EAU guidelines, especially on management related to the stone size in specific scenarios, both are generally unanimous for the majority of the principles of management.

Future studies should consider patient-reported outcomes and measurable and comparable end points, which are more homogenously reported. We also recommend that the guidelines should undergo regular updates based on recently published material, and, while these guidelines provide a framework, treatment plans should still be personalised, respecting patient preferences, surgical expertise, and various other individual factors, to offer the best outcome for kidney stone patients.

## Figures and Tables

**Table 1:** EAU and AUA recommendations based on ureteric stone size and location.

Anatomical Location	Stone Size	Type of Intervention (EAU)	Type of Intervention (AUA)
Proximal ureter	<10 mm	SWL or URS	SWL or URS
Proximal ureter	>10 mm	1. URS (first line) 2. SWL (second line)	SWL or URS
Distal ureter	<10 mm	SWL or URS	1. URS (first line) 2. SWL (second line)
Distal ureter	>10 mm	1. URS (first line) 2. SWL (second line)	1. URS (first line) 2. SWL (second line)

SWL—shockwave lithotripsy, URS—ureteroscopy.

**Table 2:** EAU and AUA recommendations based on renal stone size and location.

Anatomical Location in Collecting System	Stone Size	Type of Intervention (EAU)	Type of Intervention (AUA)
Upper/middle calyces/renal pelvis	<10 mm	1. SWL or URS (first line) 2. PCNL (second line)	SWL or URS
	10–20 mm	PCNL/URS or SWL	SWL or URS
	>20 mm	1. PCNL (first line) 2. URS or SWL (second line)	1. PCNL (first line) 2. URS or SWL (second line)
Lower pole	<10 mm	1. SWL or URS (first line) 2. PCNL (second line)	SWL or URS
	10–20 mm	SWL or URS/PCNL (favourable factors for SWL) PCNL/URS as first line, SWL as second line (unfavourable factors for SWL)	1. PCNL/URS (first line) 2. SWL (second line)
	>20 mm	1. PCNL (first line) 2. URS or SWL (second line)	1. PCNL (first line) 2. URS or SWL (second line)

SWL—shockwave lithotripsy, URS—ureteroscopy, PCNL—percutaneous neprolithotomy.

**Table 3:** Management of calcium oxalate stones.

Metabolic Assessment Outcome	EAU Recommendations	EAU Strength of Recommendation	AUA Recommendations	AUA Strength of Recommendation
Hypocitraturia	Alkaline citrate or sodium bicarbonate	Strong	Increase vegetables and fruits in diet, limit non-dairy animal protein and high-acid food Potassium citrate	Expert opinion Standard
Hyperoxaluria	Calcium supplements in enteric hyperoxaluria Pyridoxine in primary hyperoxaluria type 1 Alkaline citrate in enteric hyperoxaluria Reduced dietary fat and oxalate in enteric hyperoxaluria Oxalate restriction	Strong Strong Weak Weak Weak	Restrictive oxalate diet along with calcium supplements in case of enteric hyperoxaluria	Expert opinion
Hypercalciuria	Thiazide or alkaline citrate or both	Strong	Limit sodium intake (panel suggestion 2300 mg/day), consume 1000–1200 mg/day of dietary calcium Thiazide	Standard Standard
Hyperuricosuria	Allopurinol as first line Febuxostat as second line Avoid excessive intake of animal proteins	Strong Strong Strong	Reduce intake of non-dairy animal protein and counsel to decrease acid load and increase alkali load in diet Allopurinol	Expert opinion Standard
Hypomagnesuria	Magnesium			
Hypernatriuria	Reduce salt intake	Strong		

**Table 4:** Management of uric acid stones.

Metabolic Assessment Outcome	EAU Recommendations	EAU Strength of Recommendation	AUA Recommendation	AUA Strength of Recommendation
Hyper-acidic urinary pH < 5.5	Alkaline citrate	Strong	Potassium citrate	Expert opinion
Hyperuricosuria	Allopurinol	Strong	Reduce intake of non-dairy animal protein	Expert opinion
Urinary pH > 6.5 in ammonium urate stone	L-methionine			

**Table 5:** Management of cystine stones.

Metabolic Assessment Outcome	EAU Recommendations	EAU Strength of Recommendation	AUA Recommendation	AUA Strength of Recommendation
Cystine excretion < 3 mmol/day	Potassium citrate Increase daily fluid intake for urinary volume > 3 L/day	Strong Strong	Limit sodium and protein, increase fluid intake Potassium citrate Tiopronin, if dietary and alkalinisation therapies fail	Expert opinion Expert opinion Expert opinion
Cystine excretion > 3 mmol/day	Tiopronin in addition to above measures	Strong		

**Table 6:** Management of calcium phosphate stones.

Metabolic Assessment Outcome	EAU Recommendations	EAU Strength of Recommendation	AUA Recommendation	AUA Strength of Recommendation
Hypercalciuria	Thiazide	Strong	Limit sodium intake (panel suggestion 2300 mg/day), consume 1000–1200 mg/day of dietary calcium Thiazide	Standard Standard
Urinary pH > 6.5–6.8	L-Methionine			

**Table 7:** Management of struvite stones.

EAU Recommendations	EAU Strength of Recommendation	AUA Recommendation	AUA Strength of Recommendation
Complete surgical removal Antibiotics if persistent bacteriuria Ammonium chloride for urinary acidification Methionine as an alternative for urinary acidification Acetohydroxamic acid may be an option for persistent bacterial colonisation	Strong Strong Weak Weak	Surgical removal, and, if surgical options exhausted, acetohydroxamic acid can be offered Monitor patients with urease-producing organisms for reinfection; prophylactic antibiotics may prevent recurrence	Option Expert opinion

## Data Availability

The study data are freely available.
